# Long-term renal outcome of Cryopyrin-associated periodic syndrome (CAPS) under anti-Interleukin-1 therapy

**DOI:** 10.1038/s41598-024-67380-4

**Published:** 2024-07-18

**Authors:** Martin Russwurm, Sophia Johannsen, Birgit Kortus-Götze, Christian S. Haas

**Affiliations:** 1 Department of Internal Medicine, Nephrology and Intensive Care Medicine, Phillips-University, Baldingerstraße 1, 35043 Marburg, Germany; 2 Pharmacological Institute, Philipps-University, Marburg, Germany

**Keywords:** CAPS, Renal outcome, Long-term, Anti-IL-1 therapy, Canakinumab, Anakinra, Diseases, Nephrology, Rheumatology

## Abstract

Cryopyrin-associated periodic syndromes (CAPS) are orphan hereditary auto-inflammatory diseases with various phenotypes, including chronic kidney disease (CKD). Current therapies inhibit interleukin-1 (IL-1) to achieve clinical and serological remission; however, the effect on kidney involvement remains unclear. The objective of this study was to investigate the long-term efficacy of anti-IL-1 treatment with special emphasis on renal outcome. We retrospectively analysed clinical, genetic and laboratory data of patients with CAPS under anti-IL-1 therapy from a single-centre university outpatient clinic. Patients with CAPS (n = 28) were followed for a median of 11 (IQR 8.5–13) years. Four patients at various ages (19%), bearing the most common CAPS mutation R260W, had significant CKD at presentation. All affected patients were related; however, other family members with the same genetic variant did not develop CKD. While anti-IL-1 therapy was effective in lowering symptom burden and inflammatory parameters in all CAPS patients, two of the four individuals with significant CKD had persistent proteinuria and worsening kidney function. None of the patients without renal affection at therapy initiation developed relevant CKD in the follow-up period. We showed that in patients with CAPS: (1) CKD is a common complication; (2) renal involvement shows familial predisposition beyond the mutational status and is independent of age; (3) anti-IL-1 therapy results in sustained improvement of inflammatory parameters and symptom load and (4) may prevent development of CAPS-associated CKD but not affect kidney involvement when already present. Overall, early therapy initiation might sufficiently prevent renal disease manifestation and attenuate progression.

## Introduction

Periodic fever syndromes are autoinflammatory diseases comprising different entities characterized by recurrent episodes of fever and systemic inflammation in the absence of autoantibody production, infection or malignant disease. Cryopyrin-associated periodic syndromes (CAPS) are a subgroup of periodic fever syndromes that due to gain-of-function mutations in the NLRP3 gene result in a constitutive over-activation of the respective inflammasome^1^. These mutations eventually lead to caspase-1-mediated cleavage of pro-IL-1 into the active IL-1β. Interleukin-1β by itself drives the inflammatory response by binding to its receptor on the same cell as well as on neighbouring and remote cells. Thereby, gene expression of multiple IL-1β pathway effectors is enhanced, resulting in an autoinflammatory positive feedback loop^2^. A plethora of mutations at the NLRP3-locus has been identified, some with clear genotype–phenotype associations, yet most with uncertain significance (“low-penetrance” mutations or “variants of unknown significance / VOUS”). So far, the INFEVERS registry lists 262 mutations at the NLRP3-locus (https://infevers.umai-montpellier.fr/web/search.php?n=4).

Renal involvement in CAPS has been described to various degrees, ranging from haematuria and/or proteinuria to end-stage renal disease due to AA amyloidosis. AA amyloidosis is a typical severe complication of autoinflammatory diseases, associated with high mortality if the kidneys are affected^3^.

Anti-IL-1 therapies are the mainstay of treatment in CAPS and have been shown to be effective in both reversing symptoms and normalizing inflammatory parameters^4^. Notwithstanding, despite anecdotal evidence of improving renal AA amyloidosis^5^, data addressing long-term renal outcome under anti-IL-1 therapies remain scarce. The objective of this study was: (1) to assess frequency of renal involvement in CAPS; (2) to identify a potential association of renal outcome with genetic mutations on the NLRP3 gene; and (3) to evaluate the impact of anti-IL-1 therapy on clinical symptoms and inflammatory marker as well as renal outcome.

## Subjects and methods

In a retrospective study, patients admitted between 2001 and 2019 to the outpatient clinic of the Department of Internal Medicine, Nephrology & Intensive Care Medicine at the University Hospital Marburg, Germany, were screened for presence of an autoinflammatory disease. Patients with a diagnosis of CAPS, anti-IL-1 therapy and at least 2 follow-ups were included in data analysis. Diagnosis of CAPS was based upon clinical presentation according to expert consensus criteria^6^ or proof of CAPS-defining or related mutations and a positive family history. Exclusion criteria were other autoinflammatory syndromes or concomitant autoimmune or malignant disease.

Demographic data and baseline characteristics as well as urinary and blood parameters (serum creatinine, C-reactive protein, serum amyloid A [SAA], urinalysis, urinary creatinine, proteinuria and albuminuria) were obtained from the electronic patient data system. Where applicable, CKD stages were assigned according to the KDIGO classification^7^ based upon the estimated glomerular filtration rate using the MDRD formula (G stage, ranging from 1 to 5 with higher values indicating more severe kidney disease) and albuminuria (A stage, ranging from 1 to 3 with higher values indicating more severe urinary albumin loss). Patients with CKD stage G ≥ 3 and/or stage A > 1 were considered to have significant kidney disease. Patients treated by dialysis were marked with “D”, transplanted patients with “T”, according to KDIGO nomenclature. Symptom load that had been measured during routine patient treatment by using a clinical severity score^4^ was obtained from clinical records: In brief, nine symptom categories were assessed, comprising global assessment as general measure of well-being, skin affection, arthralgia, myalgia, cephalgia, conjunctivitis, fatigue, chills and achillodynia. A value from 1 to 5 was assigned to each symptom with higher values indicating more severity and 1 representing best well-being or absence of symptoms. The clinical severity score consists of the sum of all values; while the lowest achievable score of 9 reflects no symptoms, the highest one of 45 represents maximal symptom load. All patients had received individually adjusted dosage of anakinra or canakinumab at the physicians’ discretion. By indication, patients with renal disease received Angiotensine converting enzyme inhibitors or Angiotensin receptor blockers when proteinuria occurred and antihypertensive medication in case of arterial hypertension. Clinical and laboratory data obtained at baseline before therapy initiation and during follow-up routine outpatient visits were used to assess therapeutic efficacy. For outcome analysis, patients with and without CKD were separately evaluated.

Descriptive data are presented as mean ± standard deviation (SD) or total number and percentage. For comparison of follow-up data, a mixed-effects analysis with the Geisser-Greenhouse correction was performed. A two-sided *p* < 0.05 was deemed significant. Missing data were omitted for analyses. Correlations were calculated as Spearman’s rho (ρ). Statistical analysis was carried out using GraphPad PRISM 8 software, GraphPad Inc.

### Ethical approval

Due to the retrospective nature of the study, the medical ethics committee of the university of Marburg waived the need of obtaining informed consent. The study was thereby approved with the identifier: AZ ek_mr_20_10_2020_rußwurm. This research was carried out in accordance with the Declaration of Helsinki of the World Medical Association and complied with local guidelines for human studies.

## Results

### Baseline characteristics

**Figure 1:**
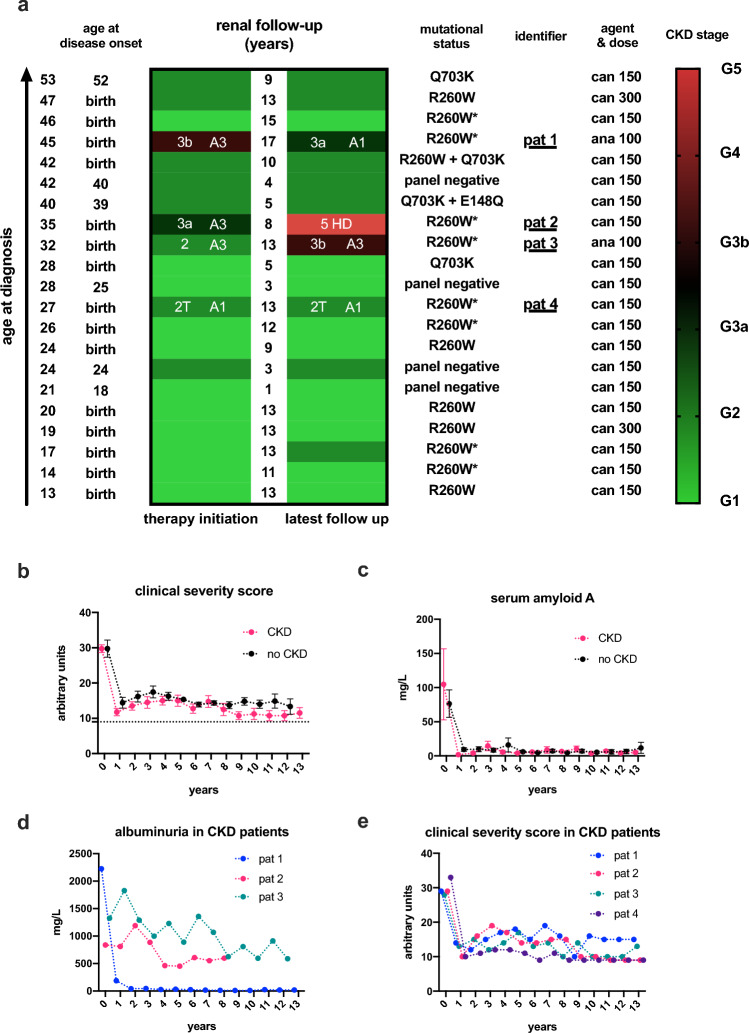
Renal disease in CAPS patients with respect to mutational status as well as clinical symptom load and inflammatory parameters under anti-IL1 therapy. (**a**) Overview of renal disease at start of anti-IL-1 therapy and at latest follow-up as defined per KDIGO stages of chronic kidney disease (CKD), with G signifying extent of eGFR (G1-G5) and A depicting amount of albuminuria (A1-A3). All patients are sorted by age at therapy initiation in descending order from top to bottom with the respective follow-up period. Additionally, individual age of symptom onset, agent and dose of anti-IL1 therapy and mutational status are depicted (please note that “E148Q” is a mutation of the MEFV gene); asterisks label the one family most frequently affected by CKD. Four patients with significant CKD are underlined and designated as *pat 1* to *pat 4.* The fourth patient (*pat 4*) was in need of renal replacement therapy at therapy initiation, and received a renal transplant 3 years later. This patient was followed for 13 years after transplantation and constantly under anti-IL1 therapy additional to standard immunosuppressive therapy for allograft rejection prevention (cyclosporine, mycophenolate mofetil and low-dose steroids). Abbreviations: can/canakinumab, administered subcutaneously every 8 weeks; ana / anakinra, administered subcutaneously every day. (**b**) Symptom burden of CKD and non-CKD patients with CAPS under anti-IL-1 therapy as measured by clinical severity score (minimum 9, maximum 45): Specific treatment resulted in significant reduction of symptom load in both groups, which was maintained over time. The dashed line represents the lowest possible score value, signifying absence of any symptoms. (**c**) Course of serum amyloid A (SAA) concentrations in CKD and non-CKD patients at therapy initiation and during follow-up: Anti-IL-1 therapy significantly and permanently lowered SAA in both groups. (d) Albuminuria of CKD patients over time showing maintained improvement of urinary albumin excretion. Data of the pat 4 is not depicted because of renal replacement therapy prior to ant-IL-1-therapy. None of the other patients presented with or developed significant albuminuria. (**e**) Individual clinical severity score (minimum 9, maximum 45) measuring symptom load in CKD patients at start of anti-IL-1 therapy and during follow-up.

Of the 28 patients included (mean age at diagnosis 30.6 ± 11.7 years; range 13–53), 19 individuals (68%) were female (Table 1). Twenty-one patients (75%) had a CAPS-associated mutation, two patients (10%) had a combination of two mutations: One patient having a R260W mutation was also bearing a Q703K mutation at the same gene locus. One patient had an additional E148Q mutation in the *MEFV* gene, which is considered as VOUS for Familial Mediterranean Fever. This patient was included into the study because of typical CAPS symptomatology and a diagnostic CAPS-score. Seventeen patients (61%) were members of four non-related families with multiple affected individuals. The CAPS-score was diagnostic in 25 patients (89%); the remaining patients had a typical symptomatology and positive family history. Of interest, myalgia and arthralgia were present in all patients (n = 28; 100%), followed by fever and rash (n = 25; 89%), while shiver and neurological sequelae were less common (43% and 29, respectively, Table 1). At time of initial presentation, all patients had a marked disease activity with a clinical severity score of 29.8 ± 1.8. At baseline, concentrations of serum inflammatory parameters SAA and C-reactive protein were also elevated in all patients. Renal parameters at baseline and follow-up were available for 21 patients. Significant kidney disease was prevalent in 4 patients (19%) at diagnosis and therapy initiation (Fig. 1a). Initial urinalysis showed proteinuria in 3 patients (14%), leukocyturia in 5 patients (24%) and haematuria in 1 individual (5%; Table 1).

**Table 1:** Baseline characteristics of all CAPS patients at time of diagnosis and prior to anti-IL-1 therapy.

		n = 28	Percentage
Gender (female/ male)		19/9	68/32
Age (years, mean ± SD; range)		30.6 ± 11.7; (13–53)	
Mutation			
Any mutation		21	75
R260W		13	62
Q703K		5	24
R260W and Q703K		1	5
Q703K and E148Q		1	5
Y482H		1	5
No mutation		7	25
Kinship single person/related persons		11/17	39/61
Family 1		8	47
Family 2		4	23
Family 3		2	12
Family 4		3	18
Symptoms			
Arthralgia		28	100
Myalgia		28	100
Fever		25	89
Rash		25	89
Headache		24	86
Conjunctivitis		23	82
Hearing loss		13	46
Shiver		12	43
Neurological symptoms, other		8	29
CAPS diagnostic score^§^			
100		19	68
52–99		6	21
0–51		3	11
Clinical severity score (mean ± SEM; range)^#^		29.8 ± 1.8 (16–45)	
Inflammatory parameters			
c-reactive protein [mg/L], mean ± SD (range) (normal < 5)		29.2 ± 3.9 (9–59)	
Serum amyloid A [mg/L], mean ± SD (range) (normal < 6.4)		83.5 ± 19.2 (7.8–247)	
Renal parameters		n = 21	
KDIGO stages	GFR [ml/min]		
G1	> 90	11	52
G2	60–89	7	33
G3a	45–59	1	5
G3b	30–44	1	5
G4	15–29	0	0
G5/RRT*	< 15	1	5
Urinalysis			
Proteinuria		3	14
Leukocyturia		5	24
Haematuria		1	5

^§^Diagnostic clinical score with a cut-off of 52 points for a diagnosis of CAPS; ^#^Clinical severity score for the disease; lowest possible value 9; highest possible value 45. Please note that the mutations “E148Q” and “Y482H” do not relate to the NLRP3 gene, but to the MEFV and PLCG2 genes, respectively. ^*^RRT renal replacement therapy.

### Outcome

Complete follow-up data were available from 21 patients with a median follow-up of 11 years (IQR 8.5–13 years; Fig. 1a). Anti-IL-1 therapy resulted in a homogenous decline in the clinical score in both CKD and non-CKD patients that was maintained throughout the study period (*p* = 0.009; Fig. 1b). Of interest, the clinical score did not differ between CKD and non-CKD patients at baseline and during follow-up. Accordingly, the significant decline in plasma concentration of SAA (*p* < 0.0001; Fig. 1c) and C-reactive protein (data not shown) was also maintained throughout the study period with no differences between both groups. Values of SAA and C-reactive protein showed a significant correlation (ρ = 0.62; *p* < 0.0001). Of note, all patients with CKD were members of one family having a R260W mutation. However, only half of the family members had renal involvement, while the other 4 family members did not have CKD at baseline nor did they develop CKD under anti-IL-1 therapy despite having the specific mutation. Nonetheless, the majority of patients of the study population did neither have CKD at baseline (n = 17) nor did they develop CKD at follow-up. Age was associated with impairment of renal function (ρ = 0.59; *p* = 0.004) in all patients. As regard to therapy regimen in individual patients, the vast majority received canakinumab at a dose of 150 mg every 8 weeks. Two patients received anakinra for patient reported lack of therapeutic effect (fatigue syndrome not resolved under canakinumab). Those two patients belonged to the CKD group, however one (pat 1) improved in CKD stage and one (pat 3) deteriorated.

All four patients with significant CKD had biopsy-proven renal amyloidosis. One patient (pat 1) had significant kidney disease with albuminuria (G3bA3) at time of CAPS diagnosis and showed improved renal function under anti-IL-1 therapy with remission of proteinuria. However, two patients (pat 2 and pat 3) with severe albuminuria had worsening CKD despite anti-IL-1 therapy (Fig. 1d). Indeed, one patient (pat 2) progressed from CKD G3aA3 to CKD G5D—formerly designated as end stage renal disease—within eight years of treatment. The other patient (pat 3) progressed from CKD G2A3 to G3bA3; both patients had persistent severe albuminuria despite anti-IL-1 therapy (Fig. 1d). The fourth patient (pat 4) had already developed CKD G5DA3 already at the age of 24, followed by kidney transplantation three years later: With anti-IL-1 therapy he remained in CKD stage G2TA1 during 13 years of follow-up. Of note, clinical symptom burden under anti-IL-1 therapy did not differ between CKD patients with favourable renal outcome and those with progressive CKD (Fig. 1e).

## Discussion

We retrospectively evaluated a cohort of patients with CAPS under long-term anti-IL-1 therapy with special emphasis on renal outcome. Patients in this study were predominantly female and tended to be older than in other studies^8–11^. Most previous studies involving CAPS patients were carried out in a paediatric population. Thus, our cohort was very different with a longer exposition to the natural course until diagnosis of CAPS and subsequently a higher risk of developing sequelae. However, as in previous reports, musculoskeletal symptoms, rash and fever were the most common symptoms in the present study^8^. As described before, inflammatory parameters and clinical symptom load improved markedly after start of anti-IL-1 therapy and remained suppressed throughout the follow-up period^10^.

Our data show that CKD is a relevant complication of CAPS, affecting 4 out of 21 patients (19%) at various ages at time of diagnosis. In an earlier study by Kümmerle-Deschner et al., frequency of renal affection in CAPS was reported to be as high as 59%^12^. In that study, however, most patients had a rather rare mutation (E311K) with very high frequency of renal involvement (77%) as opposed to patients having other mutations (47%). In addition, no patients with the R260W mutation were included, which is the predominant mutation in our study and presumably the most frequent CAPS mutation per se^8^. Notably, the authors did not define renal involvement and KDIGO criteria were not used; essentially, cut-off values of GFR and proteinuria defining CKD were not reported.

All patients with significant CKD in the present study belonged to the same family bearing the R260W mutation. Surprisingly, only 50% of family members had CKD. This finding suggests not only a familial predisposition independent of the specific disease-causing mutation but also the presence of additional factors contributing to renal injury in CAPS patients. Albeit KDIGO stages were generally associated with age at diagnosis, the risk for amyloidosis does not appear to be a simple derivative thereof, nor simply the result of the inflammatory load: In fact, the youngest patient with CKD was most seriously affected requiring renal replacement therapy from age 24 on. On the contrary, the oldest patient in that family, aged 46 at time of initial diagnosis, did not show any signs of CKD. This broad inter-patient variability in developing amyloidosis is in line with earlier reports^13^.

Anti-IL-1 therapy is the gold standard of therapy for CAPS^4^. In our cohort, it resulted in profound and sustained reduction in inflammatory parameters as well as symptom load in all patients. During follow-up, however, CKD progressed in two patients despite anti-inflammatory and clinical efficacy of anti-IL-1 therapy, while patients without significant kidney disease at therapy start did not develop CKD stages G3-5 or A3. We concluded that neither age and serum parameters before therapy initiation nor clinical or serological efficacy during therapy may predict renal outcome once CKD is present. As for the low number of patients (n = 2) in the anakinra group and the heterogenous outcomes we would refrain from inferring any conclusion regarding the specific anti-IL1 agent, especially as those patients showed homogenous therapy responses concerning serum inflammatory parameters and self-reported efficacy under anakinra.

Progress of CKD despite clinical and serological efficacy of anti-IL-1β therapy, in principle, leaves two explanatory options: First, amyloidosis might be at least in part due to a mechanism independent of IL-1 in the first place. This assumption is supported by low serum inflammatory parameters in patients with renal amyloidosis before therapy initiation but would contradict earlier findings of a landmark study on amyloidosis in autoinflammatory disease^3^. Second, and more compelling, renal disease might be already in a state of self-perpetuating damage, irrespective of primary cause. In favour of the latter argument the fact remains that in those patients no other signs of aggravating amyloid deposition were apparent in an observational period of 8 and 13 years, respectively. Furthermore, the favourable outcome of the young transplanted patient with anti-IL-1β therapy points to efficacy in preventing rather than reversing manifest amyloidosis. This assumption is finally corroborated by the fact that under therapy no new cases of CKD developed in the observational period.

The present study has some limitations, foremostly small sample size. However, despite data being per se limited to few individuals in orphan diseases, it is the first time that renal involvement in CAPS has been systematically studied. Frequency of renal affection has to be taken cautiously, especially as only a proportion of 61% of patients had identified family members with CAPS. In those families all carriers of the respective genes had been identified. However, it is likely that in the remainder cases not all gene carriers had been identified as CAPS is pre-eminently an autosomal dominant disease. This might lead to overreporting if patients with only mild symptoms might not be admitted to our clinic. Another limiting factor lies in the “unknown known” of patients not suffering from significant kidney disease in our study and who have not rigorously been examined for the presence of amyloidosis, neither by biopsy nor by SAP-scintigraphy. Hence, we cannot definitely draw conclusions, whether in those patients, subclinical amyloid deposition, if any, might be reversed or progression halted by treatment.

In conclusion, we showed that in CAPS patients: (1) CKD is relatively common; (2) development of CKD may be due to a familial risk beyond the mutational status and/or the result of a potential second hit; (3) anti-IL-1 therapy significantly improves inflammatory parameters and symptom burden; (4) development of CAPS-associated CKD may be prevented by therapies targeting IL-1, while renal outcome may not be predicted once renal damage is already present. To our knowledge, this is the first study to thoroughly investigate renal outcomes under long-term anti-IL1 therapy. This study shows for the first time, that even after initiation of anti-IL1 therapy, that is efficient as regard to serum inflammatory load and patient reported symptom burden, renal responses are heterogenous.

Overall, manifestation and progression of CKD in CAPS is possibly preventable by early therapy initiation, hence emphasizing recognition and treatment of the disease in a timely fashion.

## Data Availability

All relevant data are reported in the article. Additional data can be provided upon request by the corresponding author.
